# Comparison of machine learning models for bluetongue risk prediction: a seroprevalence study on small ruminants

**DOI:** 10.1186/s12917-022-03486-z

**Published:** 2022-11-09

**Authors:** Hagar F. Gouda, Fardos A. M. Hassan, Eman E. El-Araby, Sherif A. Moawed

**Affiliations:** 1 Department of Animal Wealth Development, Faculty of Veterinary Medicine, Zagazig University, Zagazig, 44511 Egypt; 2 Department of Animal Wealth Development, Faculty of Veterinary Medicine, Suez Canal University, Ismailia, 41522 Egypt

**Keywords:** Random forests, Classification, Variable importance, Machine learning, Bluetongue, ANN, Logistic regression, Small ruminants

## Abstract

**Background:**

Bluetongue (BT) is a disease of concern to animal breeders, so the question on their minds is whether they can predict the risk of the disease before it occurs. The main objective of this study is to enhance the accuracy of BT risk prediction by relying on machine learning (ML) approaches to help in fulfilling this inquiry. Several risk factors of BT that affect the occurrence and magnitude of animal infection with the virus have been reported globally. Additionally, risk factors, such as sex, age, species, and season, unevenly affect animal health and welfare. Therefore, the seroprevalence study data of 233 apparently healthy animals (125 sheep and 108 goats) from five different provinces in Egypt were used to analyze and compare the performance of the algorithms in predicting BT risk.

**Results:**

Logistic regression (LR), decision tree (DT), random forest (RF), and a feedforward artificial neural network (ANN) were used to develop predictive BT risk models and compare their performance to the base model (LR). Model performance was assessed by the area under the receiver operating characteristics curve (AUC), accuracy, true positive rate (TPR), false positive rate (FPR), false negative rate (FNR), precision, and F1 score. The results indicated that RF performed better than other models, with an AUC score of 81%, ANN of 79.6%, and DT of 72.85%. In terms of performance and prediction, LR showed a much lower value (AUC = 69%). Upon further observation of the results, it was discovered that age and season were the most important predictor variables reported in classification and prediction.

**Conclusion:**

The findings of this study can be utilized to predict and control BT risk factors in sheep and goats, with better diagnostic discrimination in terms of accuracy, TPR, FNR, FPR, and precision of ML models over traditional and commonly used LR models. Our findings advocate that the implementation of ML algorithms, mainly RF, in farm decision making and prediction is a promising technique for analyzing cross-section studies, providing adequate predictive power and significant competence in identifying and ranking predictors representing potential risk factors for BT.

## Background

Bluetongue (BT) is an infectious, noncontagious, viral disease transmitted by insects and affects wild and domestic ruminants, predominantly sheep [1]. The disease is a major concern for livestock, resulting in direct losses from deaths, abortions, lower meat and milk production, and high disease control and prevention costs. Export restrictions on live animals and their products cause indirect losses [2]. The World Organization for Animal Health estimated global economic losses at $3 billion [3]. Improving BT diagnostic tools, surveillance, and vaccination with circulating serotypes are viable ways to control the disease, which is difficult to eradicate due to subclinical cases, prolonged viremia, and the spread of vectors that keep the virus in the environment [4].

Machine learning (ML) is a recently emerging subfield of computer science. Previously, data analysis relied primarily on traditional methods, which can’t handle large amounts of data perfectly, particularly in case of outliers [5]. Fortunately, ML has overcome these shortcomings. ML updates accurate prediction models through self-learning. Categorical and numerical variables can be analyzed. ML can deal with high-dimensional data and with nonlinear problems [6].

The rationales for ML and traditional methods differ. Statistical models begin with model hypotheses, set the assumptions, and focus on inference. ML is primarily concerned with forecasting; the best-performing predictive model is chosen. ML's hypothesis-free nature makes it an appealing choice for complex data analysis [7]. High-quality ML data used in the training of farm-decision-making models improves model prediction accuracy and prevents overfitting [8].

The use of ML algorithms for predicting veterinary risk factors is still uncommon. Few studies have used ML algorithms to investigate BT risk factors*.* In this study, we developed prediction models to investigate and assess BT-associated risk factors as an adjunct to improving veterinary health and disease screening in five Egyptian governorates. This study aims to address small data applications of ML models for classification tasks with the objectives of (1) developing ML prediction methods, including random forests (RF), decision trees (DTs), artificial neural networks (ANNs), and logistic regression (LR), producing BT results as binary output (i.e., BT or non-BT) and class probability using various BT-related variables; (2) evaluating the models’ performance and examining the contribution of variables to BT classification; and (3) performing validation of the BT prediction models to assess their reliability.

## Results

### Comparison of models’ performance

Table (1) presents the performance of the four ML algorithms in the training and testing phases. In the training phase, the models performed differently; RF and ANN achieved the highest accuracy (85.5%) and AUC (up to 85%). As expected from our theoretical motivations and results, ML algorithms provided evidence of their better predictive ability than LR in the testing set. Moreover, we observed that the classification provided by RF and ANN was much more balanced in terms of accuracy, TPR, precision, and AUC than LR. RF and ANN showed the best predictive model ability and no effect of overfitting in metrics. The model estimated by ANN was the nearest in performance to RF. DT recorded the lowest FPR (13%) and best precision (69%) among all models, but the highest FNR (47%) and the lowest TPR (53%), confirming that one tree has a much lower discriminative power of classes than many trees (RF). Although DT and LR's metrics fluctuated, their prediction performance was quite close. Overall, ML algorithms outperformed LR in the training and testing sets.

**Table 1:** The results of the training and testing subsets of the four algorithms

	**Training data**	**Testing data**
**1. RF**		
**Accuracy**	85.5 (0.80–0.90)	83 (0.69–0.92)
**TPR**	0.78	0.77
**FPR**	0.08	0.15
**FNR**	0.22	0.23
**Precision**	0.88	0.67
**F1 score**	0.82	0.71
**AUC**	0.85	0.81
**2. DT**		
**Accuracy**	78.5 (0.72–0.84)	74.5 (0.60–0.86)
**TPR**	0.74	0.53
**FPR**	0.18	0.13
**FNR**	0.26	0.47
**Precision**	0.78	0.69
**F1 score**	0.76	0.60
**AUC**	78.4	0.73
**3. ANN**		
**Accuracy**	85.5 (0.79–0.90)	81.00(0.67–0.91)
**TPR**	0.80	0.77
**FPR**	0.10	0.18
**FNR**	0.20	0.23
**Precision**	0.86	0.63
**F1 score**	0.83	0.69
**AUC**	84.89	79.6
**4. LR**		
**Accuracy**	75.00 (0.68–0.81)	72.00 (0.57–0.84)
**TPR**	0.64	0.61
**FPR**	0.16	0.24
**FNR**	0.36	0.38
**Precision**	0.75	0.50
**F1 score**	0.69	0.55
**AUC**	0.74	0.69

*TPR* True positive rate, *FPR* False positive rate, *FNR* False negative rate, *AUC* Area under the curve, *RF* Random forest, *DT* Decision tree, *ANN* Artificial neural network, *LR* Logistic regression

### Analysis of variable importance

**Fig. 1:**
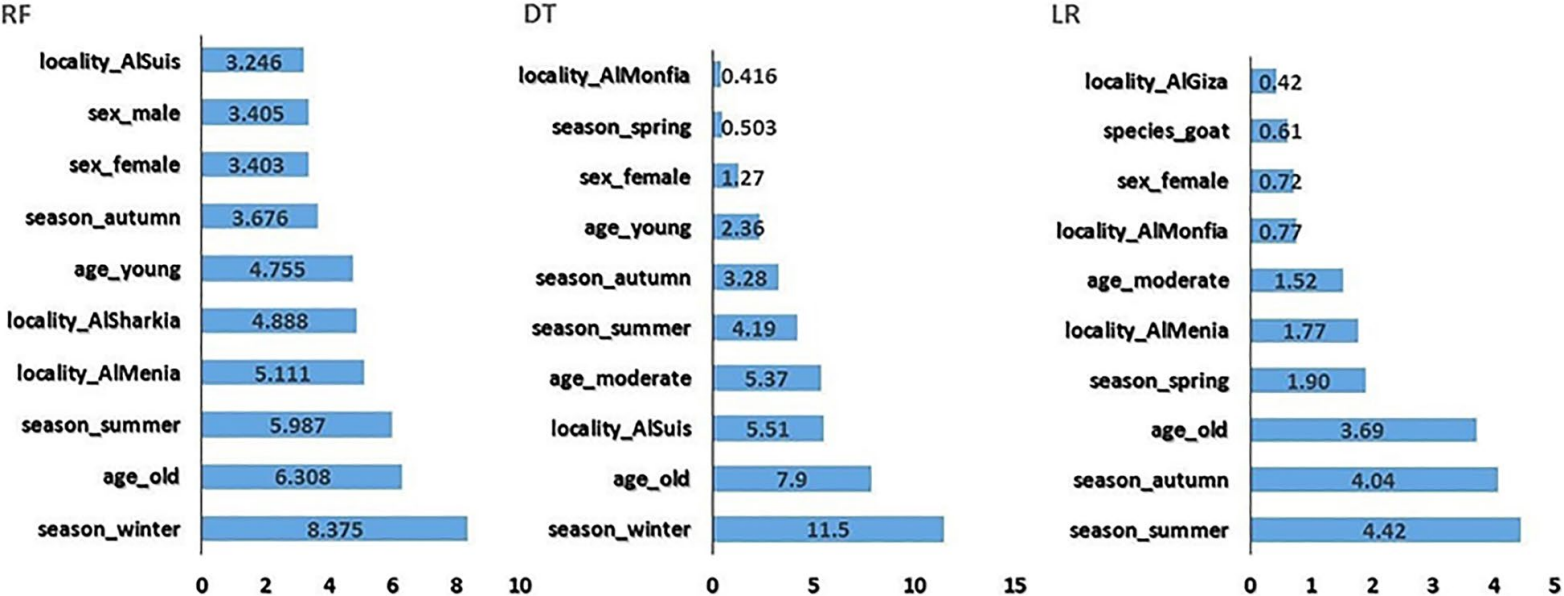
The top-ranked variables and their corresponding importance values for random forest (RF), decision tree (DT), and logistic regression (LR)

**Fig. 2:**
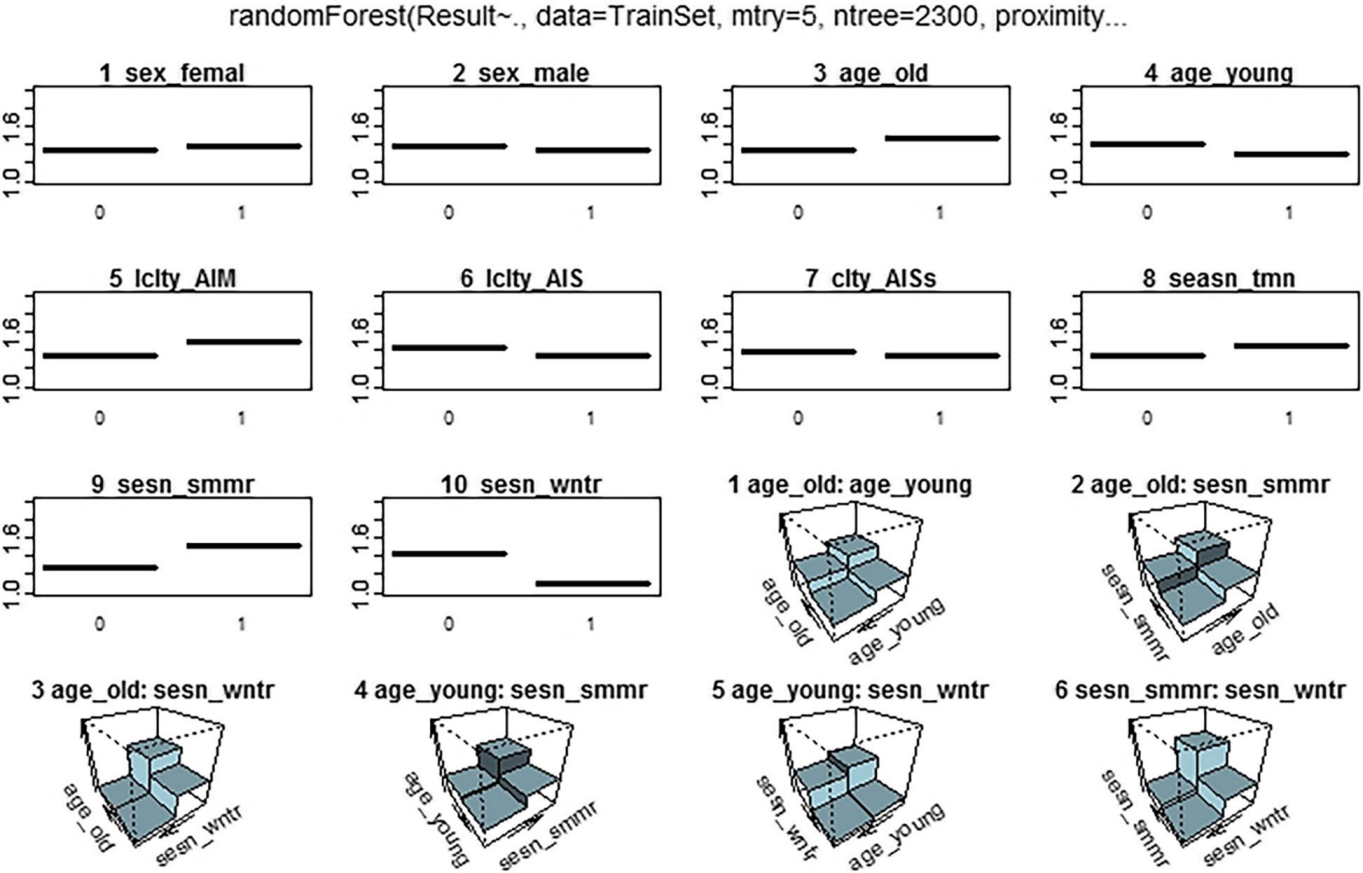
Partial dependence plots of RF for the top selected predictors. Since the predictors are dummy coders, 1 denotes a class with the given variable name and 0 denotes the other classes of the variable

**Fig. 3:**
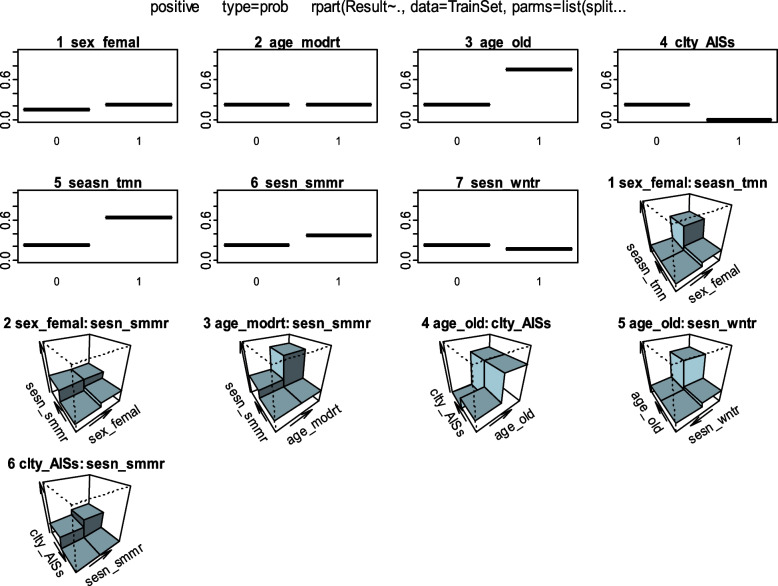
Partial dependence plots of DT for the top selected predictors. Since the predictors are dummy coders, 1 denotes a class with the given variable name and 0 denotes the other classes of the variable

**Fig. 4:**
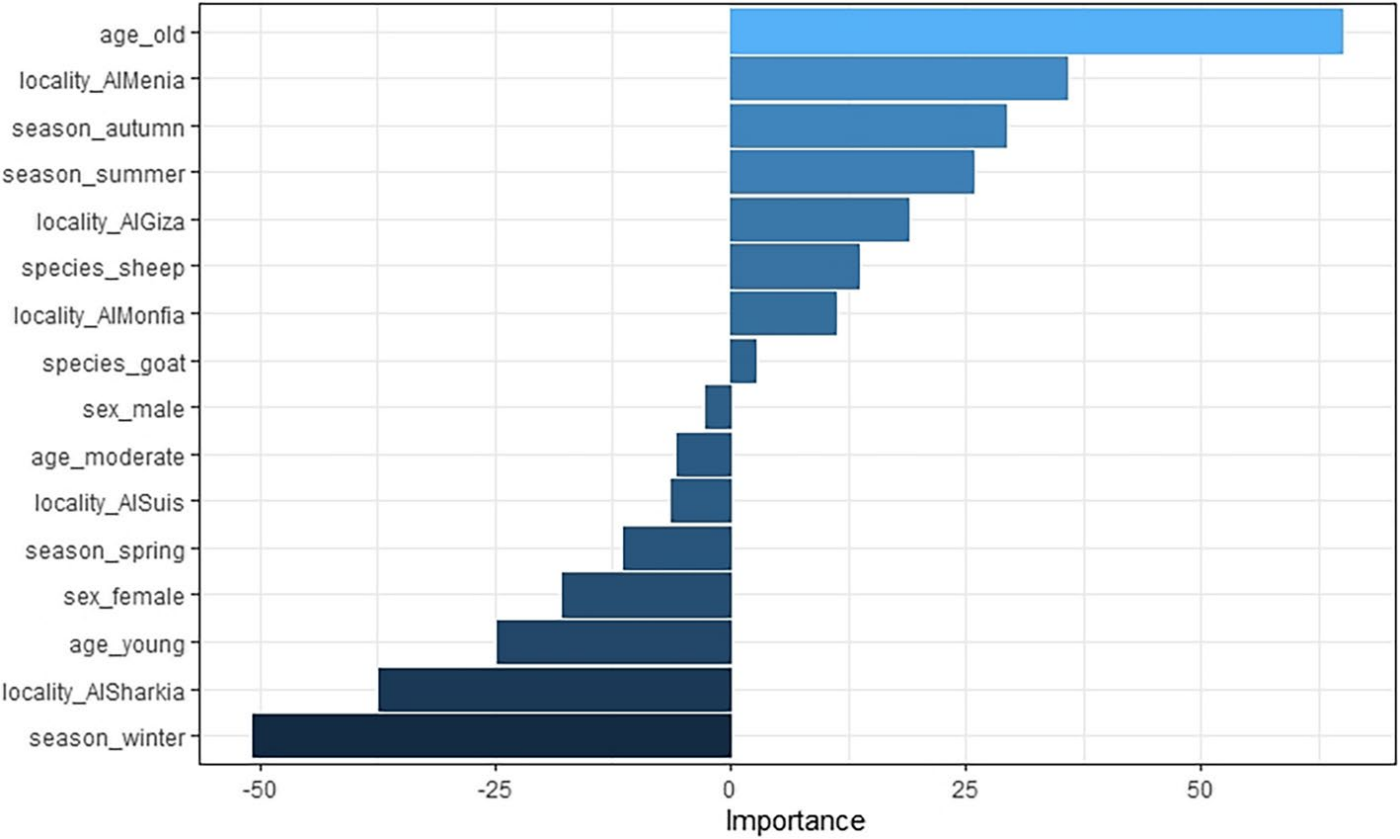
Relative variable importance of ANN model, created by Olden function. The bars on the right indicate the variables with a positive impact on increasing BT risk, while the bars on the left indicate those with an inverse effect

Figures (1, 2, 3, and 4) present the top 10 variables based on the variable importance of each algorithm. The season and age of the animals played the most crucial role in BT classification in all models. Old-age animals, autumn, and summer are top-ranked by all four algorithms as positively related to BTV. Conversely, young-age animals and/or winter were related to seronegative BTV.

## Discussion

To the best of our belief, our study was the first, which applied and compared ML methods to assess and predict BT risk in Egypt, and epidemiologically, this is one of the few studies in veterinary medicine that will be scrutinized. We developed prediction models based on a seroprevalence study to predict BT risk factors using DT, ANN, RF, and LR models. Because of advances in biotechnology and information technology, ML application is straightforward [9]. Several papers [10, 11] have provided evidence for using ML in the medical field to increase the likelihood of successful ML applications and overcoming challenges. Unlike the traditional methods, we were not required to formulate hypotheses about the ML models we used, nor were we constrained by the data distribution.

The acceptable accuracy of ML algorithms in clinical studies significantly increases their application compared with conventional methods, such as LR [12]. Romero et al. [13] used the RF and LASSO regression models to predict bovine tuberculosis. Model accuracy increased (up to 29%) when class imbalance was treated with repeated cross-validation and down-sampling. Giannuzzi et al. [14] applied several ML models to select the best model for predicting the cow blood metabolic profile using the milk. Gradient boosting machine and ANN outperformed RF, stacking clusters, and other regression models among the techniques used. In a study conducted by Mota et al. [15] to predict cheese-making traits in Holstein cattle, they compared the prediction of ANN, elastic net, gradient boosting machine, and extreme gradient boosting, and ANN achieved the highest predictions.

The RF ML method has been effectively applied in several fields, including veterinary science [16]. Although few veterinary epidemiological studies adopt ML-based algorithms, most of these studies ignore the importance of tuning model parameters [17, 18].

The predictors used in our study were all categorical and dummy coded; Loh and Vanichsetakul [19] recommended dummy coding of the categorical variables. This has another impact in that the Gini variable importance measure is strongly biased because it allocates greater importance to variables with extra categories or continuous variables [20]; so, to avoid this issue, we used dummy coding of categorical predictors. No evidence of collinearity of the predictors detected in our results. Although RF stands for variable collinearity, some studies have shown that correlated variables are preferable in the process of growing trees [21]. Applied ML models were trained on 80% of the data tested and evaluated on the remaining 20%. We had to ensure that our models were not prone to over-or under-fitting because we built them on a small data set. Five repeats of tenfold cross-validation were used on training data to improve the models’ estimated performance and tuning hyperparameters, and the best performing parameters were selected to build the model on the entire training set without the cross-validation split. An independent testing set was used to evaluate the model. Some studies have recommended using five or ten-fold cross-validation when the sample size is greater than 200 to accommodate both the variance and bias of the model [22, 23]. However, Molinaro et al*.* [24] suggested using repeated k-fold cross-validation or Monte Carlo cross-validation (MCCV) when the sample size is small, for the advantage of less susceptibility to high variance, and increasing the number of repeats lowers the variance without elevating the bias. Differently, Song et al*.* [25] proposed leave-one-out cross-validation (LOOCV) as an appropriate choice for a very small sample size.

The performance metrics of the four ML models' training and testing sets were compared. According to Jiang et al. [26], comparing the measurements of the two groups is important for the model's generalization by analyzing the generalization gap. Therefore, the performance measures in the training and testing sets should be as close as feasible. Otherwise, there might be two issues: overfitting with greater measurements in the training set and poor in the testing set, and underfitting with measures too low in the training set to be generalized [27]. Our findings revealed that RF and ANN maintained their good performance in the testing set compared to the training set, while DT and LR didn’t. The RF accuracy was higher than that of a single DT, which is consistent with a previous study [16].

Few studies have compared the efficacy of ML classification for predicting BT risk. A previous study [28] applied RF to identify the most prominent factors for BT. The study recorded an AUC of (86%) and a TPR of (75%). These results are comparable to our results. RF recorded an AUC of (81%), and a TPR of (77%).

Regarding the results of the variable importance analysis, we discussed only the most relevant variables. RF, ANN, DT, and LR demonstrated that the most prominent predictors were old age, summer, and autumn. The age predictor was previously reported as a significant factor in the prediction of BT in studies [29], which also revealed that older animals were more vulnerable to BT than younger ones. By contrast, Yavari et al*.* [2] discovered that the rate of seropositivity decreased in old-age animals. This is probably due to the more frequent exposure of the animals to BT infection. Alternatively, Nayel et al. [30] reported an insignificant effect of age on BT risk in goats, whereas a significant association (OR > 1) was observed in the case of sheep.

RF, DT, and LR showed females as an important predictor related to BT seropositivity. These findings are consistent with a previous study [29].

The algorithms take the season into account while predicting BT risk. Purse et al. [31] discovered that when temperatures range from 20 °C to 30 °C, the risk of disease propagation increases considerably due to an increase in the number of culicoides. Otherwise, the cooler the temperature is, up to 12.5 °C, the less likely illness will spread [32]. Our findings show that summer and autumn are strongly associated with increased BT risk, but winter and spring are not. Similarly, El-Bagoury and Moneer [33] discovered that autumn had the highest incidence of BT (21.1%). In contrast, Alzuheir et al. [34] found that the incidence of BT was lowest in the spring and summer (May–June). Based on ANN variable importance, BT risk in sheep is higher than in goats. The findings are consistent with a prior study [35]. The results of RF, ANN, and LR show a positive impact of Al-Menia government on BT risk. ANN and DT indicate that Al-Giza governorate is of positive concern. These findings are consistent with Mahmoud et al. [36].

## Conclusions

In this study, we compared ML techniques for improving BT risk prediction using small-scale data. The study findings revealed that ML approaches outperform LR. The need for further ML application in veterinary epidemiology was underlined, which might improve herd health and help veterinary decision-making, resulting in improved clinical treatment and increased profitability by keeping the disease under control. Overall, RF performed best, with the highest accuracy, AUC, and F1 score, closely followed by the ANN. Although DT had the lowest FPR, it showed the highest FNR, indicating inferior stability and predictive performance. Finally, LR was of poor quality, with markedly more deficient accuracy, precision, and F1 score than the other ML techniques utilized in the study. We may infer that while ML algorithms can handle multidimensional data, they demonstrate a promising impact in implementing prediction and classification using small datasets (233 in our study). LR may perform better with a larger sample size in epidemiological or clinical investigations.

## Methodology

Four ML algorithms were applied to predict BT: RF, ANN, DT, and LR. LR was used as a baseline for performance comparison as it is a classic regression algorithm that focuses on binary classification problems. The performance of the models was assessed in terms of accuracy, TPR, FPR, FNR precision, F1 score, and AUC. These metrics ranged from 0 to 1. AUC measures the discriminative power of the model between presence/absence classes. For example, an AUC value of 0.5 means that the model is not better than a random guess; values 0.5–0.7 indicate poor discrimination, 0.7–0.8 acceptable, 0.8–0.9 excellent, and 0.9–1 exceptional discriminative power [37].

### Data source

Materials and methods used the data of a seroprevalence study conducted on 233 (125 sheep and 108 goats) apparently healthy animals. Samples were collected from April 2018 to March 2019 from five governorates in Egypt.

### Diagnostic test

Competitive ELISA has been carried out for the detection of antibodies against BTV VP7 antigen in serum samples of sheep and goats using ID Screen Bluetongue competition kit (ID VET, Grabels, France). According to instructions of the manufacturer, a positive reaction is scored when the percent of OD sample/OD negative control (S/N %) is less than 40%. Of all examined animals, 40.3% were seropositive, whereas 59.7% were seronegative.

To classify the status of the animal disease, we used classification models $$\widehat{y}$$(***x***) trained on a labelled set of training examples, $${\{{y}_{i},{x}_{i}\}}_{i=1}^{N}$$. Each of the ***N*** examples denotes an animal, where ***x***$$\in$$$${\mathbb{R}}^{d}$$ is a d-dimensional vector of predictors, and y $$\in$$ {0, 1} is the animal’s outcome, encoded as 1 if the BT is diagnosed and 0 otherwise. We used **X** to refer to a matrix of predictors/features with ***N*** rows and ***d*** columns. Table (2) lists the predictors and the outcome.

**Table 2:** Demographic variables used in ML models for BT prediction

**Variables**	**Description**	**Type of variable**
**Predictors:**		
**1. Species**	Sheep or goat	Categorical
**2. Age**	Young (6- < 18 months), moderate (18- < 36 months), and old (≥ 36 months)	Categorical
**3. Sex**	Male and female	Categorical
**4. Governorate**	Al-Sharkia, Al-Monfia, Al-Menia, Al-Giza, and Al-Suis	Categorical
**5. Season**	winter, spring, summer, and autumn	Categorical
**Outcome:**		
**Bluetongue**	Positive or negative serotyping	Categorical

All predictors are categorical in nature and were dummy coded into 0 and 1, so we have 16 input variables

### Data preparation

The selection of risk factors was performed by chi-square test. Variables with a significant level (p < 0.05) were considered important and kept for supervised classification. All variables showed a significant relationship with BT. Data were randomly assigned to a training dataset (80%) for model creation and a testing dataset (20%) for prediction performance verification. We used five repeats of ten-fold cross-validation on the training data to train the model and tune the hyperparameters. The tuned hyperparameters were subsequently used to build a more accurate predictive model. Finally, based on the metrics in the testing set, the ML model that showed the best discrimination and accuracy was determined to be used in predicting BT.

### Decision tree

The DT is a statistical model that aims to make an accurate prediction that minimizes the loss function, which measures the differences between observed and expected values. The model uses a set of factors to predict the response based on several hierarchical binary divisions of the data in the form of a tree, hence the name. Successive data splits produce subsets of data, and in each subset, the expected result is obtained by averaging the results of all individuals in the subset [38]. Common splitting criteria include information gain, Gini index (GI), and gain ratio. Classification and regression trees (CART) [39] is the most common form of DT application.

This easily understood hierarchical graphical structure of the DT makes it a reliable predictive approach to support decision-making [40]. Moreover, DT does not necessitate tuning many parameters in the design [41].

**Fig. 5:**
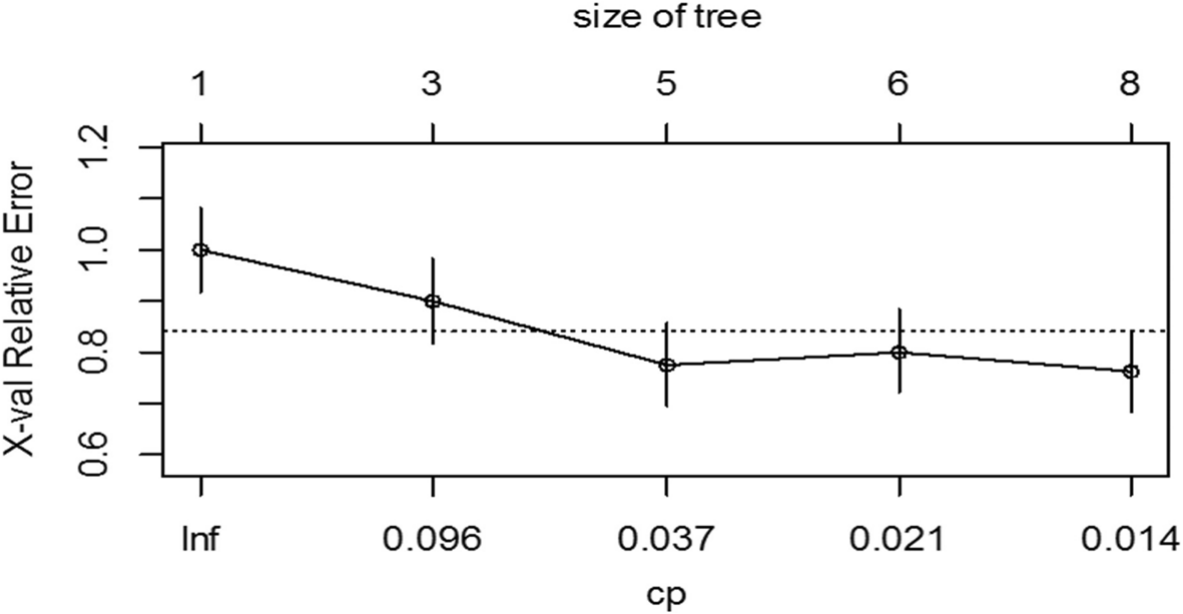
The tree size in relation to cost-complexity parameter (CP) and cross-validation error (x-val Relative Error), the dashed horizontal line represents one standard deviation of the minimum cross-validation error. The tree pruned after 7.^th^ split, CP = 0.01

In this study, DT was constructed with the rpart function in the “rpart” package in R, using GI as the criterion for selecting attributes and achieving the best overall split. The minimum size for the split was set to 25, the minimum number of observations in the terminal node was 7, and the maximum depth was 30. Based on the cross-validation (x-Val = 10) results of the base model, DT was pruned by specifying a complexity parameter (CP). Figure 5 shows that a CP value of 0.01 corresponds to a tree with seven splits (eight nodes) with xerror = 0.76543 > (relative error = 0.49383 + xstd error = 0.07937). This value of CP = 0.01 will be used to prune the classification tree.

### Random forest

RF algorithm is a predictive statistical model consisting of a set of DT obtained through random and independent selection of data subsets. Predicting a class in RF is simply based on getting a majority vote for all trees. The RF is simple as it requires neither the data distribution assumptions nor the relationship between the predictors and the target variable. RF is also resistant to autocorrelation and multicollinearity issues [42]. RF has been considered one of the most precise predictive models in categorical and numerical data analyses, including classification, regression, and unsupervised learning, with significant advantages, including variable importance detection and handling of the interaction of predictors [43]. The RF produces an unbiased classification model with lower susceptibility to overfitting in case of unbalanced data distribution [44]. It contains more than one crucial hyperparameter that should be optimized to avoid the potential problems with the algorithms. The number of trees (ntree), number of predictors selected at each split (mtry), and tree size are the most tunable hyperparameters to consider, and the effect of mtry tuning is of paramount importance [45]. The RF features used for training parameters in this study were (i) ntree = 2300 and the number of trees generated (ii) mtry = 5.

It has a notable advantage of revealing the variable importance, an indicator that shows the impact of each variable used in the prediction [42]. Gini and permutation variable importance are two of the most commonly used measures [20].

### Artificial neural network

ANN is an ML model that mimics the machinery of how the human brain works. It can handle complex biological data. Unlike traditional statistical methods, it is characterized by the ability to quickly identify data patterns (linear and nonlinear) and contingency effects [46].

Multilayer perception (MLP) is a simple form of ANN that in most conditions, comprises three successive layers: the input layer that contains the data to be processed, the output layer that performs the classification task, and, in the middle, a hidden layer that is the engine of any ANN computational procedure [47].

The backpropagation algorithm is the most common ANN training algorithm, and it has two phases: feedforward and feedback. The feedforward phase determines the output, whereas the feedback phase calculates the error and updates the weights. By adjusting the weights, the error between the actual values and the ANN output is gradually reduced [48].

This study used a feedforward backpropagation neural network with a sigmoid activation function. Additionally, a sigmoid function was used at the hidden layers to introduce nonlinearity to the network for learning and allowing the algorithm to generate complex mappings between the features and the outputs.

The activation function often used in feedforward neural networks is the sigmoid function [49].

The ANN model has 16 neurons in the input layer, two in the output layer, and 11 in the hidden layer. The appropriate number of hidden layer neurons is determined based on the experiment till achieving a sufficient error reduction, as there is no established theory for number setting yet. The optimal number of hidden layer neurons generates a better predictive neural network model. Tuning parameters included the number of nodes in the hidden layer optimized between 1 and 15. The threshold used was 0.07, and the learning rate was 0.001. Finally, the neurons in the input and the hidden layers were activated using the sigmoid function. The error function was cross-entropy. The error was 47.86. Nevertheless, it required 31,842 steps for convergence in 9.72 s.

### Logistic regression

LR is a statistical method used to assess the relationship between a binary target variable and one or more predictors of any measurement level [50]. To determine the relationship between BT-infected animals and the potential risk factors, a LR model was developed.

### Data analysis

All analyses were performed using R Statistical Software v.4.1.1 [51]. CART was constructed using the “rpart” package (v.4.1.16) [52], “randomForest” [53] for RF, “caret” [54] for training models, “neuralnet” for ANN [55], and “glm” in the “stats” package for LR classifier. For variable importance of RF, we performed a variable importance analysis that assesses the average decrease in node impurity measured by the GI, “Importance” function from the “randomForest” package, and “vi” function from the “vip” package for DT. Furthermore, to determine which variables are significant for the positive BT class, a partial dependence plot was constructed, which presents a graphical depiction of the marginal effect of a variable on the class probability (Fig. 2) for RF and (Fig. 3) for DT. Greater y-values indicate that observation for a definite variable is associated with a higher probability of classifying new instances as BT positive. The “Olden” function from the “NeuralNetTools” package [56] was used to measure the relative importance of input variables in neural networks as the sum of the product of raw input–hidden, hidden–output connection weights, as proposed by Olden et al. [57]. Finally, the importance of variables in LR was assessed by effect sizes and p-values of the Wald or likelihood ratio test [45].

## Data Availability

The datasets used and analyzed during the current study available from the corresponding author on reasonable request.

## Abbreviations

BT: Bluetongue
LR: Logistic regression
DT: Decision tree
RF: Random forest
ANN: Artificial neural network
AUC: Area under the curve
ML: Machine learning
TPR: True positive rate
FPR: False positive rate
FNR: False negative rate
CART: Classification and regression tree
GI: Gini index
CP: Complexity parameters
MLP: Multilayer perceptron
LOOCV: Leave one-out-cross-validation
MCCV: Monte Carlo cross-validation
